# Improvement of Attention in Elementary School Students through Fixation Focus Training Activity

**DOI:** 10.3390/ijerph17134780

**Published:** 2020-07-03

**Authors:** Yi-Jung Lai, Kang-Ming Chang

**Affiliations:** 1 Department of Early Childhood Educare, Wu Feng University, Chiayi 62153, Taiwan; yrlai@wfu.edu.tw; 2 Department of Photonics and Communication Engineering, Asia University, Taichung 41354, Taiwan; 3 Department of Medical Research, China Medical University Hospital, China Medical University, Taichung 40402, Taiwan

**Keywords:** focus training activity, elementary school children, Attention Scales, focus attention, selective attention

## Abstract

The attentional problems of school children are a crucial topic due to abundant information in this digital era. There are five attention dimensions for children: focused attention, sustained attention, selective attention, alternating attention, and divided attention. Focused training is a traditional method of improving attention ability. Subjects are required to focus on a fixed point for an extensive period without blinking and to perceive small objects as large. This study investigates which types of attention indicators are influenced by focus training. Eighty-two grade five and six elementary school students (45 experiment group, 37 control group) were involved. The experiment group underwent focus training for 12 weeks. The training was conducted once per week, and the Attention Scales for Elementary School Children were used before and after the training to examine the children’s attention. The percentile rank scores of five attention dimensions and the total attention scale were evaluated. The results gave difference data, defined as post-test results minus the pretest results, where significant differences occurred for the total scale (*p* < 0.05), focused attention (*p* < 0.05), and selective attention (*p* < 0.01). Participants also noted that the training helped them improve concentration during school lessons (54.15%), fall asleep (29.1%), and relax the body (8.4%).

## 1. Introduction

Amid the current rapid circulation of information and the proliferation of digital products, information sources available to students are more abundant than ever, and this reduces the attractiveness of conventional learning. In classroom learning and afterschool reading, the performance of school-age children has worsened. Parents often attribute this to reduced attention. Therefore, the attentional problems of school children are a crucial topic. Theories and opinions have been developed in academia regarding attention in school-age children. Among these theories, the “clinical model of attention” proposed by [1] divides attention into five dimensions:

(1) Focused attention refers to an individual’s ability to directly respond to specific visual, auditory, or tactile stimuli. Sohlberg and Mateer asserted that focused attention impairment typically occurs in the early stages of recuperation from a coma for patients who sustained brain injury and that focused attention is often the earliest recovered attentional function for such patients.
(2) Sustained attention is the ability to maintain consistent behavioral responses during continuous or repetitive activities. Individuals who experience impairment in this attentional dimension can only focus transiently on a task or maintain a response for several seconds or minutes. They may also exhibit dramatic fluctuation in sustained attention over a short period of time.
(3) Selective attention is an individual’s ability to maintain a behavior or cognitive set when faced with distractions or competing stimuli. Patients deficient in this attentional component can be easily distracted by stimuli that are irrelevant to their original task. These irrelevant stimuli generally include various types of external distractions (stimuli from the external environment such as scenery, sounds, or activity) or internal distractions (an individual’s internal worries, thoughts, or contemplations of personal importance to the individual). Clinically, patients with selective attention deficiency often cannot undergo therapy in a place with other stimuli present.
(4) Alternating attention is an individual’s ability to change the focus of attention and the mental flexibility to shift between tasks with different cognitive requirements. Patients with alternating attention deficiency have difficulty changing from a familiar stimulus–response model. They often require extra prompts to cope with changes of task. This attentional dimension is critical for students, such as when shifting between listening to lectures and writing notes. The cognitive requirements for the two tasks are different. Therefore, students must rely on their mental flexibility to effectively alternate their attention.
(5) Divided attention is an individual’s ability to simultaneously respond to multiple tasks. Individuals engaged in divided attention handle tasks simultaneously across multiple stimuli (e.g., listening to the radio while driving or talking to others while preparing a meal). Under these circumstances, the individual must execute alternating attention rapidly and continuously or rely on subconscious automated procedures to manage one of multiple tasks.

The main methods available for measuring attention include using relevant instruments [2,3] and using questionnaires [4]. To measure the attention of elementary school children in Taiwan, Taiwanese researchers designed the Attention Scales for Elementary School Children, which form the only standardized rating scale with local (Taiwanese students) norm data. The scored norms of the scale were established by grade level. The established norms include the dimensional score and percentile rank of the five attentional dimension subscales and the attention index norms and percentile rank norms of the total scale. As well as the reliability and validity data from the Attention Scales for Elementary School Children, additional data were collected using the Wechsler Intelligence Scale for Children, Third Edition, Freedom from Distractibility Index and the multi-dimension attention test to verify the criterion-related validity of the scale. The reliability and validation of the scales used is listed in Appendix A. The Attention Scales for Elementary School Children form a multidimensional attention measurement scale that links attention theories with attention training programs. In addition to providing references for teachers and medical personnel to objectively screen elementary school students for attention deficits, the testing results obtained using the Attention Scales for Elementary School Children can help medical personnel clarify the direction of subsequent focus training and ameliorate attentional problems in students. Respondents have 30 min to complete this scale, which is divided into ten subtests, and every two subtests correspond to an attention indicator. A briefing is usually provided to the respondents for 10 min before filling out the scale. Currently, this scale is used extensively to measure the attention of elementary school students in Taiwan [5].

**Figure 1:**
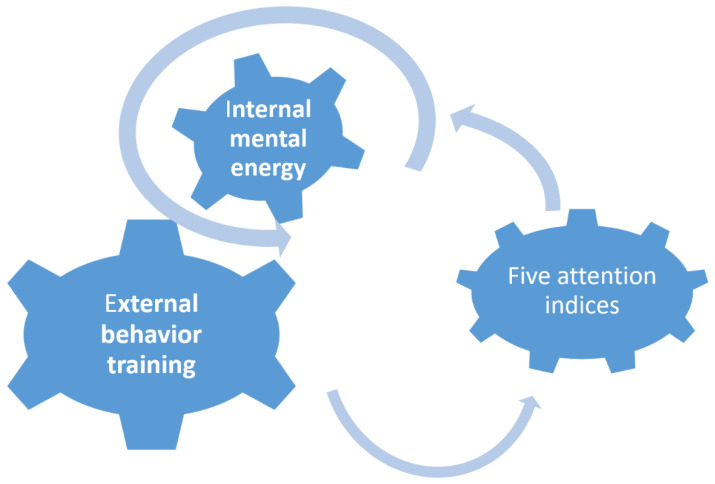
Attention training model.

The Attention Scales for Elementary School Children facilitate the use of numerous focus training methods for systematic evaluation. Some common pediatric focus training methods include gaming (e.g., table games), solving maze puzzles, engaging in hands-on creation (e.g., playing with blocks, cooking, and playing with clay), and participating in sports activities (e.g., archery and rope skipping) [6,7]. Meditation and mindfulness meditation are focus training methods that have been popular in recent years [8,9,10]. External focus training has been used since ancient times. With this training method, concentration power and willpower are enhanced by focusing on a fixed point for an extended period of time. This method was also described in an article entitled *Ji Chang Learning Archery* within the *Tangwen* chapter of the ancient Chinese book *Liezi*. Two emphases of the training were described in the article: focusing on a fixed point for an extensive period without blinking and perceiving small objects as large. The field of vision should only contain the item focused on [11]. Training methods to improve students’ attention include computer games [12], neurofeedback [13,14,15], virtual and augmented reality [16,17], music [18], and meditation [19]. Table 1 provides details of these methods. As shown in the table, training methods have involved guiding students to immerse themselves in activities or design tools, such as games. Each method focused on one specific attention index. However, with the exception of meditation, all methods involved focus on objects outside one’s own body rather than on oneself. Attention must be coordinated with changes in one’s own behaviors, such as conscious activities. Subconscious activities, such as reflexes and autonomic nerve-regulated activities, also affect attention. There are two ways to improve the attention index: one is by external behavior training, such as games or task operation; the other is by focus and the improvement of internal mental energy, such as by meditation. This model is shown in Figure 1. Which attention index will be reflected by internal focus training? Will all metrics improve? To investigate these issues, this study focused on the discussion of the following questions:

Q1: Can the attention indices of elementary school students be improved through attention training?
Q2: Is the ancient Chinese attention training approach, which focuses on changes to the inner state of mind, truly effective for improving the attention of elementary students? If yes, which attention indices are improved?

In the experiment, a training model recorded in ancient Chinese literature was employed to train elementary students, and the changes in the attention indices were recorded.

**Figure 2:**
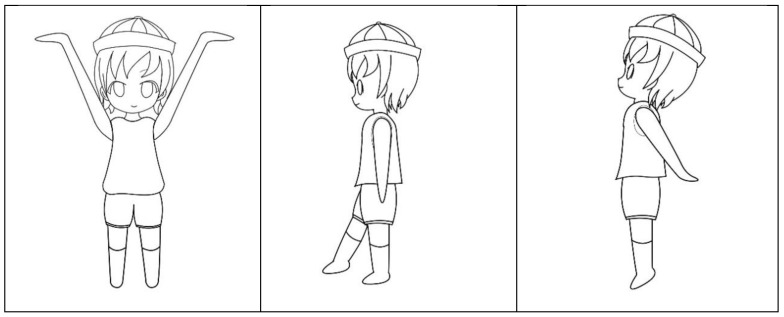
Illustration of dynamic focus training actions.

The focus training method in the current study is similar to the aforementioned methods. The participants must undergo one training session per week for 12 weeks, and each training session spans approximately 1 h. During the training, participants focus their vision on a fixed point, such as the heart, palm of the hand, or fingertips. Several examples of the training activity are illustrated in Figure 2. Before this study, the distribution norms of adults’ external focus index were already established by using eye trackers to record the pupil focus trajectory of many adults [11]. During measurement, the participants only needed to fixate on the screen for 1 min and were instructed to fixate on the internal area of a circle. In addition, the participants were not in contact with any device or equipment. The current study examined the effect of focus training on the attention of elementary school students by administering the Attention Scales for Elementary School Children. In addition, we identified and examined the types of attention indicators that were influenced by focus training.

## 2. Materials and Methods

### 2.1. Subject Information

A pretest–post-test research design was employed. All participants were from the same elementary school. After consent was obtained from the school authority, explanations of the experiment were distributed to fifth- and sixth-grade students, who relayed these to their parents. The students joined either the experimental or control group or declined to participate in the experiment subject to their parents’ approval and their own free will. None of the teachers intervened in the explanations or encouraged or prevented the students’ participation. All decisions regarding participation in the experiments were made by students and their parents. The participants were divided into experimental and control groups. After the participants were briefed on the experiment details, they were asked to fill out the participant and parental consent forms before the experiment began. The scale was administered to participants of the same grade level in groups. All participants were from the same elementary schools. Table 2 presents the participant information.

### 2.2. Experiment Design and Focus Training

The participants in the experimental and control groups were tested together in batches according to their free time. In addition to demographic data, other data were collected from responses to the Attention Scales for Elementary School Children and participants’ performance in focusing on the eye-tracking device for 1 min. The eye-tracking results were analyzed and discussed separately from the data collected from the scale. Subsequently, the experimental group underwent focus training for 12 weeks. The training was conducted once per week, and each training session spanned approximately 60 min. Two weeks after the experiment was completed, the Attention Scales for Elementary School Children was administered in groups to obtain the post-test results. The procedures of the focus training are described as follows:

Step 1. Sit still for approximately 3 min and wait for all students to arrive.
Step 2. Before the activity commences, remind participants to fixate on a specific point (or figure) on the wall in front of them.
Step 3. Begin the dynamic focus training (approximately 20 min). Throughout the process, consistently remind the students to focus on a fixed point on the wall in front of them.
  Action 1: Throw both hands backwards while standing on the toes and slightly tilt the head backwards. Keep attention on the heart.
  Action 2: Kick two legs forward in alternation and maintain balance in the upper body while keeping it still. Keep attention on the waist.
  Action 3: Bend the knees slightly and shake the whole body. Keep attention on the abdomen near the umbilicus.
  Action 4: Keep the body straight and squat slightly. The hands should be kept on both sides of the body, and the palms should be pressed down while parallel to the ground. Keep attention on the knees.
  Action 5: Stretch the hands upward vertically and stretch the whole body. The palms should be stretched upward while parallel to the sky. Keep attention on the face.
  Action 6: Stretch the hands upward diagonally in a relaxed manner. The two arms should form an approximately 60° angle. Keep attention on the lower heart.
  Action 7: Clasp hands together, put them in front of the chest, and squat deeply. Keep attention on the lower abdomen.
  Action 8: Walk on the tiptoes without bending the knees. Keep attention on the spine.
  Action 9: Tap the back of the head using the fingers while clenching the teeth. Keep attention on the middle of the head.
  Action 10: Slap the abdomen using both hands alternatively. Keep attention on the digestive system.
Step 4. After the dynamic training is completed, perform static focus training (approximately 10–15 min). Invite the students to sit down cross-legged. Their hands should be placed on their knees, their back should be straight, and their eyes should be shut lightly. First, invite the students to breathe naturally. After the students’ breathing is stabilized, request that the students focus their attention on different body parts according to the teacher’s instruction. The sequence of focus is the heart, stomach, perineum, caudal vertebrae, lumbar vertebrae, cervical vertebrae, vertex, between the eyes, and the throat. After the students are led through the focus training procedures a few times, they are given the chance to focus on body parts on their own according to this sequence.
Step 5. Conduct an experience-sharing and combined discussion session.

Some of the actions are illustrated in Figure 2.

### 2.3. Parameter Extraction from the Attention Scales for Elementary School Children

**Figure 3:**
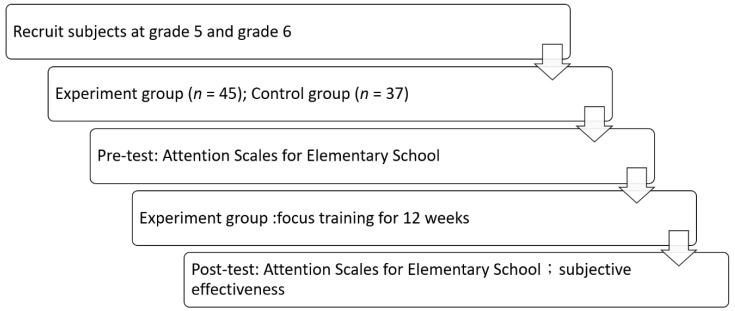
Experiment flowchart.

The raw scores for the five attention categories calculated using the questionnaire were converted into normative data and ultimately converted into grade-equivalent percentile rank scores. High percentile ranks indicated a high level of attention. The same weightage was assigned to the percentile rank scores of the five attention subscales, and the percentile rank of the total scale could be obtained by adding these together. Therefore, six types of percentile rank parameters were obtained in this study: total scale, focused attention, sustained attention, selective attention, alternating attention, and divided attention. After the post-test was completed for the experimental group, those participants were asked to provide their subjective effectiveness feedback regarding the focus training. The feedback data were organized and classified using keywords. A flowchart of the experiment is shown in Figure 3.

### 2.4. Statistics

Statistics on the pretest, post-test, and pretest–post-test difference (i.e., post-test minus pretest) are presented as mean and standard deviation. Two types of statistical tests were conducted:

(a) The paired sample *t*-test compared within-group differences between the pretest and post-test results of the experimental and control groups.
(b) The *t*-test analyzed differences between the experimental and control groups. Three sets of data were analyzed: pretest, post-test, and the post-test–pretest difference.

In addition, experiential feedback from the participants regarding the training course was organized and is displayed using keywords and percentages.

These experimental procedures were reviewed and approved by the Institutional Review Board of China Medical University (Institutional Review Board number: CRREC-107-051)

## 3. Results

The results of the Attention Scales for Elementary School Children questionnaire are displayed in Table 3. The results of P2 indicate that the experimental and control groups differed nonsignificantly in questionnaire scores during the pretest, which indicated that the attention distribution of the experimental and control groups before training was similar. For the total scale, the mean pretest percentile rank of the experimental group was 46.07, whereas the mean pretest percentile rank of the control group was 51.21. The mean pretest percentile rank for both groups was located near the midpoint (i.e., 50 points) of the total normative scores. Therefore, the participants for this study were well chosen. The lack of extremely high or low attention scores indicated that the chosen sample was representative of students of the same age.

The P1a and P1b data reveal that nearly all attention indicator scores were higher in the post-test than in the pretest, and that this was true for both groups. To understand if the focus training conducted resulted in significant changes in the experimental group, the DIFF value was defined as each participant’s post-test results minus the pretest results. The P4 results indicated that when testing the DIFF, significant differences occurred for the total scale, focused attention, and selective attention. For the total scale, the mean DIFF value of the experimental group was 27.20, and this was the attention parameter category in which the experimental group exhibited the greatest increase. However, the mean total scale DIFF of the control group was 18.92, and the mean sustained attention DIFF of the control group was 27.79. This was the category in which the control group exhibited the greatest increase. For the focused attention items, the mean DIFF of the experimental group was 18.07, whereas that of the control group was 5.24. The change in the scores for the experimental group was significantly greater than that of the control group. For the selective attention items, the mean DIFF of the experimental group was 20.38, and that of the control group was 8.32, and the difference was statistically significant (t = 2.473, *p* < 0.01). Selective attention is the indicator with the most prominent significant difference between the experimental and control groups.

The subjective feedback of the experimental group was divided into five categories. The students noted that they experienced improved concentration during school lessons, fell asleep faster at night, felt more relaxed, and experienced less stress, and some even experienced alleviated chest pain following the training. The ratio of each experience is presented in Table 4. Improved concentration during school lessons was the most frequent feeling experienced by the participants, followed by falling asleep faster at night.

## 4. Discussion

This study employed a focus training program, in which participants focused on a fixed point for an extended period. Both dynamic and static focus training sessions were provided, which required the participants to focus their attention. The statistical test results regarding pretest–post-test differences indicated that focused attention and selective attention in the experimental group improved significantly. This finding was consistent with the objectives of the training methods used in this study.

Focused attention is an individual’s ability to directly respond to specific visual, auditory, or tactile stimuli.

Selective attention is an individual’s ability to maintain behavior or cognition when faced with distractors or competing stimuli.

These two attentional components related to the focus training methods used in this study. The participants were required to maintain their focus on a fixed point on their body for a short time. The participants needed to eliminate other distractors, regardless of whether they were performing dynamic focus training or static focus training, and this is selective attention. Focused attention is the fundamental ability to respond to stimuli. Thus, the focus training method designed in this experiment could effectively improve the focused attention and selective attention of the students, and this was reflected by the total scale. Other research has indicated that selective attention involves a selection process that allows individuals to select and ignore irrelevant stimuli from their environment [20]. In the current age of information saturation, humans face a tremendous amount of information daily. Therefore, how the human brain makes decisions is a key topic. Selective attention helps determine the level of importance of external stimuli. Thus, stimuli of little importance can be deleted before the brain further processes them. Selecting stimuli from an extremely complex and ever-changing environment laden with multiple emotions is influenced by many factors. As well as the physical characteristics of the stimuli, factors such as humans’ personal interests, motivations, and cognitive strategies for receiving stimuli affect the attentional selection process [21]. Since selective attention involves filtering external information, it is crucial for human learning and development. The focus training method used in the current study could improve focused attention and selective attention in normally developing elementary students, and this can contribute positively to the learning and development of elementary students.

As shown in Figure 1, there are two major attention training models: external stimulation using interesting games [12,14,16,17], and the use of biofeedback to enhance the effectiveness of concentration exercises [13,14]. On the other hand, the most relevant research for this focused training is mindfulness meditation training [8,9,10,19]. This study belongs to the mode of internal observation. This training method is similar to the mindfulness meditation method. In Kabat-Zinn’s study, mindfulness meditation was described as a process of attending experiences of the present moment with an open, accepting, and non-judgmental attitude [22]. The focus training method used in this study was derived from an ancient Chinese book, and is widely used by modern Heart Chan meditation practitioners. The training method they use cultivates internal mental energy. Practitioners feel both mental and physical improvement.

In addition to examining the influence of the focus training intervention on the attention indicators of elementary school students (by using the Attention Scales for Elementary School Children), sorting subjective feedback from the participants revealed an interesting phenomenon: focus training appeared to improve physical and mental health. Feedback provided by the students revealed that after training sessions, the students could fall asleep more easily at night, felt relaxed, and felt less stressed. These indicated emotional improvement and an autonomic nervous regulation effect of the focus training. Some other studies have obtained similar results. An earlier study by [23] revealed that participants’ heart rate decreased after meditation, which was also found to enhance heart rate variability. Meditation has also been found to improve sleep [24] and can regulate emotions [25]. The static component of the focus training method of the current study was partially similar to meditation. Therefore, future research can examine if focus training improves the emotions and sleep of elementary school students. Both groups exhibited improvement in their post-test scores compared with their pretest scores. As indicated in Appendix A, the retest reliability of the survey is around 0.71–0.91, with a retest interval of four weeks. This shows that the results of the tests before and after the questionnaire are highly reliable. Therefore, this phenomenon should be related to changes in the subject.

This was possibly because before the post-test, the participants already understood the questionnaire content due to the pretest; therefore, filling in the questionnaire responses was easy, which ultimately resulted in higher scores.

In this study, we preliminarily explored the effect of focus training on elementary school students’ attention indicators. The participants corresponded to the general norm, and patients with special conditions were not considered. Future studies can include participants in different conditions (e.g., participants with insomnia, learning disabilities, and mood disorders) in their examinations. In addition, the results of this study indicate that focus training is related to selective attention, which is related to working memory [26]. Future studies can observe students’ working memory before and after focus training. In addition, selective attention is related to the recent perceptual load theory. Research has indicated that, regardless of whether the subject is an adult or child, the timing of selection is influenced by the level of a task’s perceptual load. The level of perceptual load depends on the difficulty of executing a task. When executing a complex task with a high difficulty, the perceptual load sustained by the human body rises and vice versa [27]. Whether focus training improves tasks’ perceptual loads merits exploration. Focused attention may relate to the human brain [28,29]. By using neuroscience equipment such as electroencephalography or functional magnetic resonance imaging, future research can examine changes in brainwave signals under the influence of ongoing focus training, which may be beneficial for emotional regulation and sleep. Focus training performance in students with mood disorders and changes in students’ sleeping patterns in the early, middle, and late stages of training would be interesting research directions in the near future. In addition to elementary students, high school and university students should be included in this model for further analysis of its effects on students in various academic phases.

This study has numerous key implications for clinical and health practices. The attention training model employed in this study has been confirmed to be stable; teachers can apply this model in all elementary school grades after receiving relevant training in teaching techniques. Moreover, no special teaching facilities or tools were required at the site of the training, and each activity required only 40–50 min. This training method is applicable for flexible courses or for morning time in elementary schools. The training model also involves meditation, which calms and relaxes students, effectively improving their selective attention and enhancing their learning capacity.

Some details should be noted in the experiment. The experiment should be conducted in a classroom where people can sit on the floor, eliminating the need for tables and chairs and reducing noise that interrupts the class. The experiment should also ideally be conducted during the morning before formal classes, preventing interference in formal classes as well as enhancing effectiveness. The experiment requires a teacher qualified in the training of students’ attention as well as a teaching assistant. The teacher should also be equipped with a microphone and speakers with clear sound quality. Furthermore, support from the administrative branch enables the effective execution of the training course during the semester; this facilitates consistent practice among students, which is a primary factor in the success of attention training.

This experiment was subject to limitations. Students’ attention had to be elicited at the beginning of the experiment. After the first few weeks of consistent practice, students should experience benefits from their training and subsequently be able to practice independently. This requires a qualified teacher. The training teacher in this study was the principal author of this study, who has interacted with students for a long time and is familiar with their personalities. The author also has several years of experience in attention training and is familiar with the physical and psychological improvements resulting from training. Furthermore, prior to the present study, the author was involved in many cases of elementary school student attention training. The greatest limitation of this study was the challenge of training a teacher to lead attention training. Future studies should investigate the number of hours required to train elementary school teachers as qualified attention training facilitators as well as the appropriate training course contents. Although the attention index scale applied in this study was suitable for elementary students in Taiwan, it is not an internationally accepted scale. Future studies should adopt various other questionnaires or instruments for the measurement of changes in students’ attention.

## 5. Conclusions

In this study, the Attention Scales for Elementary School Children were used to measure changes in elementary school students before and after 12-week focus training activities. The results revealed that the experimental group exhibited more favorable performance in all three examined indicators, namely focused attention, selective attention, and the total scale. Participants who underwent training also noted that the training helped them fall asleep and relax the body.

## Figures and Tables

**Table 1:** Summary of related studies.

Ref No.	Researcher	Training Method	Corresponding Attention Indices	Participants	Country/Region	Year
[6]	Mahar	Physical activity	N.A.	8 and 9 years	USA	2011
[7]	Schmidt et al.	Physical activity	d2-R, focused attention	11 years	Switzerland	2016
[8]	Crescentini et al.	Mindfulness-based interventions	No significant decrease in SMFQ scores (Short Mood and Feelings Questionnaire)	7–8 years	Italy	2016
[9]	Tarrasch	Mindfulness-based interventions	Higher proportion of correct trials in the Stroop task	2nd, 4th, and 6th grades	Israel	2017
[10]	Mak et al.	Mindfulness or meditation interventions	At least one outcome measure of attention	Review article	Australia	2017
[12]	Cerezo et al.	Tabletop games	N.A.	ADHD	Spain	2019
[13]	Shereena et al.	Neurofeedback	Sustained attention, verbal working memory, and response inhibition	ADHD	India	2019
[14]	Liu et al.	Fingertip-based adaptive force control tasks	Attention network test and response time	22 and 26 years	China	2019
[15]	Kosmyna et al.	Biofeedback glasses	N.A.	N.A.	USA	2019
[16]	Shema-Shiratzky et al.	Virtual reality training	Executive function and memory	ADHD	Israel	2019
[17]	Lorenzo et al.	Augmented reality	N.A.	Autism	Spain	2018
[18]	Barbaroux et al.	Music training	Visuomotor Precision test (NEPSY-II)	7 to 12 years	France	2019
[19]	Sprawson et al.	Mindfulness meditation	HEXACO-60-PI	18 to 33 years	UK	2020

N.A., not available; ADHD, attention deficit hyperactivity disorder.

**Table 2:** Subject information.

Item	Experimental Group	Control Group
Total subject number	45	37
Male/female	22/23	13/24
Number of grade 6 students (12 years old) /number of grade 5 students (11 years old)	36/9	37/0

**Table 3:** Questionnaire results. The percentile rank data are represented as mean, SD (in parentheses), maximum (in bold parentheses, []) and minimum percentile rank (in angle brackets, <>).

Attention Type	Experimental Group				Control Group				Experimental Group vs. Control Group		
	Pre-Test	Post-Test	DIFF	P1a	Pretest	Post-test	DIFF	P1b	Before P2	After P3	P4 DIFF
Total scale	46.07 (29.28) [96.0] <3.0>	73.27 (26.64) [99.0] <4.0>	27.20 (20.39) [68.0] <1.0>	***	51.21 (27.83) [95.0] <1.0>	70.13 (25.01) [98.0] <1.0>	18.92 (16.74) [50.0] <−14.0>	***			*
Focused	45.13 (29.33) [98.0] <4.0>	63.20 (29.05) [99.0] <4.0>	18.07 (31.84) [86.0] <−80>	***	49.16 (25.68) [99.0] <0.1>	54.40 (25.64) [98.0] <0.1>	5.24 (21.80) [54.0] <−38.0>				*
Sustained	36.65 (28.83) [97.0] <0.2>	61.03 (33.12) [99.0] <0.2>	24.37 (25.19) [78.0] <−18.0>	***	42.22 (26.90) [95.0] <0.2>	70.00 (23.65) [99.9] <0.2>	27.79 (17.76) [65.0] <0>	***			
Selective	70.20 (26.44) [99.0] <9.0>	90.58 (15.10) [99.0] <20.0>	20.38 (22.95) [87.0] <−11.0>	***	73.26 (26.04) [99.0] <3.0>	81.58 (24.44) [99.0] <1.0>	8.32 (21.14) [67.0] <−23.0>	*		*	**
Alternating	58.19 (28.14) [99.0] <0.4>	74.91 (27.78) [99.0] <0.1>	16.73 (20.32) [76.0] <−41.9>	***	52.45 (27.04) [94.0] <0.1>	60.79 (27.69) [97.0] <1.0>	8.34 (25.91) [72.0] <−53.0>	*		*	
Divided	36.57 (27.49) [90.0] <0.1>	51.52 (31.22) [96.0] <0.2>	14.95 (21.61) [60.0] <−26.0>	***	40.01 (26.36) [96.0] <0.2>	61.29 (28.20) [96.0] <0.2>	21.29 (23.46) [64.0] <−21.0>	***			

P1a and P1b are paired *t*-test results for the experimental and control groups. P2, P3, and P4 are the statistical testing results between the pretest, post-test, and pretest–post-test results from the experimental and control groups. DIFF—Each participant’s post-test results minus the pretest results. * *p <* 0.05, ** *p <* 0.01, *** *p <* 0.001.

**Table 4:** Experiences and statistics from training course.

Experience	Improved Concentration during School Lessons	Falling Asleep Faster at Night	Feeling Relaxed	Feeling Less Stress	Alleviated Chest Pain
Percentage	54.1%	29.1%	8.4%	4.2%	4.2%

Data were obtained from subject feedback from the experimental group after the post-test. The subjects were asked to express their experience and their changes due to the focus training course. The feedback data were organized and classified using keywords.

## Appendix A

Internal consistency of the test scores (α): 0.77–0.83; internal consistency of the subscales: 0.73–0.92; retest reliability (retest interval of four weeks): 0.71–0.91; subtest internal correlation: 0.31–0.94; subscale internal correlation: 0.39–0.80; correlation with the multidimensional attention test: 0.65; correlation with the attention indices in the Wechsler Intelligence Scale for Children, third edition: 0.53; correlation with academic grades: 0.34–0.64.
